# Dietary fiber intake and risk of gallstone: a case–control study

**DOI:** 10.1186/s12876-023-02752-0

**Published:** 2023-04-11

**Authors:** Asal Neshatbini Tehrani, Saeede Saadati, Zahra Yari, Amin Salehpour, Amir Sadeghi, Ghazal Daftari, Moloud Ghorbani, Azita Hekmatdoost

**Affiliations:** 1 Student Research Committee, Ahvaz Jundishapur University of Medical Sciences, Ahvaz, Iran; 2 Department of Nutrition, School of Allied Medical Sciences, Ahvaz Jundishapur University of Medical Sciences, Ahvaz, Iran; 3 Department of Medicine, School of Clinical Sciences, Monash University, Melbourne, Australia; 4 Department of Nutrition Research, Faculty of Nutrition Sciences and Food Technology, National Nutrition and Food Technology Research Institute, Shahid Beheshti University of Medical Sciences Tehran, West Arghavan St. Farahzadi Blvd., Sharake Qods, Tehran, Iran; 5 School of Public Health, Occupational Health Research Center, Iran Universityof Medical Sciences, Tehran, Iran; 6 Research Institute for Gastroenterology and Liver Diseases of Taleghani Hospital, Shahid Beheshti University of Medical Sciences, Tehran, Iran; 7 School of Medicine, Tehran University of Medical Sciences, Tehran, Iran; 8 Department of Community Nutrition, Faculty of Nutrition and Food Sciences, Tabriz University of Medical Sciences, Tabriz, Iran; 9 Clinical Nutrition and Dietetics Department, Faculty of Nutrition Sciences and Food Technology, National Nutrition and Food Technology Research Institute, Shahid Beheshti University of Medical Sciences Tehran, Tehran, Iran

**Keywords:** Gallstone, Dietary fiber, Cholelithiasis

## Abstract

**Background:**

Gallstone disease (GSD) and its complications are major public health issues globally. Although many community-based studies had addressed the risk factors for GSD, little is known about the associations between dietary factors and risk of disease. The present study aimed to investigate the potential associations between dietary fibers with the risk of gallstone disease.

**Methods:**

In this case–control study, 189 GSD patients with less than one month of diagnosis and 342 age‑matched controls were enrolled. Dietary intakes were assessed using a 168-item semi-quantitative validated food frequency questionnaire. Crude and multivariable-adjusted hazard ratios (HRs) and 95% confidence intervals (CIs) were estimated through cox proportional hazards regression models.

**Results:**

Comparing the highest versus the lowest tertile, significant reverse associations were observed between odds of GSD and each category of dietary fiber intake including total (OR _T3 vs. T1_ = 0.44, 95% CI: 0.37–0.7, P for trend = 0.015), soluble (OR _T3 vs. T1_ = 0.51, 95% CI: 0.3–0.8, P for trend = 0.048) and insoluble (OR _T3 vs. T1_ = 0.56, 95% CI: 0.3–0.9, P for trend < 0.001). The relationship between dietary fiber intake and the risk of gallstones was more prominent in overweight and obese subjects than in subjects with a normal body mass index.

**Conclusion:**

Comprehensive assessment of the associations of dietary fiber intake with GSD showed that higher intakes of dietary fiber were significantly associated with reduced GSD risk.

## Background

Gallstone disease (GSD) is a major public health concern with a worldwide prevalence of 2–20% [1, 2]. Age and gender are two crucial factors in the prevalence of GSD. Compared to younger age, the prevalence of GSD increases more than 10 times in men and women over 60 and 50 years, respectively [3]. In addition to genetic predisposition, there are several pathogenic factors related to GSD, including increased biliary mucin secretion, hepatic hypersecretion of cholesterol and supersaturated bile, faster cholesterol crystallization and gallbladder stasis [4, 5]. Furthermore, there is an association between GSD and parameters related to metabolic syndrome such as obesity, type 2 diabetes and dyslipidemia [6, 7].

Recently, rising attention has been addressed to detect the association of dietary factors and GSD. It has been reported that high prevalence of gallbladder diseases can be explained by increased intake of sugar and fat and decreased intake of whole grains and high-fiber foods [8]. Moreover, a cohort study of 5000 women participating in National Health and Nutrition Examination Survey indicated that the increased risk of GSD-related hospitalization following dieting could be due to reduced dietary fiber intake [9]. Westernized diets (low-fiber, high-refined carbohydrate, high-fat) have also been shown to be associated with an increased risk of gallstones [4]. Fiber may have protective effects against gallstones by reducing the intestinal transit time and reducing the production of bile acids [10].

Since detecting the protective dietary factors is important in the management of GSD, thus, the purpose of the present study is to explore the association of dietary fiber and gallstone risk.

## Methods and materials

This case–control study was conducted in the Research Institute for Gastroenterology and Liver Diseases of Taleghani hospital affiliated to Shahid Beheshti University of Medical Sciences, Tehran, Iran. Subjects with the age of 18 years and older, eagerness to participate, had approved GSD and a month or less passed from GSD diagnosis were considered to be included in the present study. Pregnant and lactating mothers and subjects with a history of intestinal disorders, autoimmune diseases, cancers, inflammatory and infectious diseases were excluded. Controls were matched to cases regarding the age (± 5 years) and sex. Patients admitted to other departments of the same hospital with no history of GSD and other liver ailments confirmed by ultrasonography, were randomly allocated to control group. It is worth mentioning that hospital controls are preferred over community controls when the cases obtained from the hospitals [11]. Because, the intended exposures such as health problems and debilitated behaviors of this type of controls are more likely to be similar to hospital cases. In fact, the prevalence of diseases in hospital controls is higher than that of the population-based controls [12]. Considering the exclusion of 5 cases with outrange mean energy intake (± 3SD) and subjects with uncompleted data, 189 cases and 342 controls (*n* = 531) remained for analyses. The protocol of the present study was approved by Research Institute of Gastroenterology and Liver Diseases Ethics Committee (IR.SBMU.RIGLD.REC.1396.159). All participants signed a written informed consent.

### Dietary intake assessment

A valid reproducible semi-quantitative food frequency questionnaire (FFQ) [13] was used in order to determine the food intake of cases and controls before GSD diagnosis and hospital admission respectively, during a previous year. The frequency of food intake for each subject described on daily, weekly, monthly or yearly basis. The collected data were analyzed using Nutritionist IV software. The United States Department of Agriculture (USDA) food composition table (FCT) was used to calculate energy and nutrient contents. In addition to total fiber, the contents of insoluble and soluble fiber were calculated and expressed as gram per day.

### Data collection

Information on socio-demographic factors, anthropometric measurements and other variables like physical activity, comorbidities, smoking habit and alcohol consumption in previous year collected by a trained interviewer through face-to-face interview. Participants body weight were measured to the nearest 100 g [14] while standing on digital scales (Soehnle, Berline, Germany). Measurement of height was done by a portable non-stretch meter to the nearest 0.5 cm. Body mass index (BMI) was calculated by division of weight in kilograms to square of height in meter. Data for physical activity was acquired by a valid questionnaire and described as metabolic equivalents hour per day (METs h/d). MET levels included in this questionnaire ranged from light (0.9 METs) to high-intensity activities (> 6 METs) [15, 16].

### Statistical analysis

Statistical analysis was performed by SPSS software version 19 (SPSS Inc., Chicago, Illinois). We used the Kolmogorov–Smirnov test and histogram chart to check the normality of variables. In the present study, we described participant’s baseline characteristics and dietary intakes as mean ± SD for quantitative variables and numbers (percentage) for qualitative variables. Independent sample t-test and chi-square were applied for determining the differences between cases and controls for variables with normal distribution and categorical variables, respectively. Subjects were classified into tertiles regarding each category of fiber intake including total, insoluble and soluble fiber. *P*–value for the trend of GSD risk across each category of fiber intake was assessed using linear regression test. Occurrence of GSD and some of its risk factors including BMI, age and sex were illustrated across tertiles of each category of dietary fiber intake. The association between dietary fiber intakes with the odds of GSD was calculated using logistic regression. The analysis was adjusted for potential confounders including age and sex, energy intake, BMI, physical activity, smoking, and alcohol consumption. The odds ratio (OR) with 95% confidence interval (CI) of GSD across tertiles of each category of dietary fiber intake were reported in regard to some of the GSD risk factors including BMI, age and sex. *P* values < 0.05 were considered statistically significant.

## Results

General characteristics of subjects and their dietary intakes are shown in Table 1. Patients diagnosed with GSD had higher mean age, consumed more fat and more likely to be female (*P* < 0.05) but they had less physical activity, total and insoluble dietary fiber intake (*P* < 0.05) than the control group (Table 1).

**Table 1:** Baseline general characteristics and dietary intakes of study participants

	Total (*n* = 531)	Case (*n* = 189)	Control (*n* = 342)	*P* value
Men, n (%)	202 (38%)	55 (29%)	147 (43%)	0.002
Age (y)	53 ± 13	55 ± 15	52 ± 12	0.005
Alcohol drinker	13 (2.4%)	6 (3.2%)	7 (2%)	0.559
Smoker, %	78 (15%)	31 (16%)	47 (14%)	0.443
IPAQ level, %				< 0.001
1	400 (75%)	174 (92%)	226 (66%)	
2	115 (22%)	10 (5%)	105 (31%)	
3	16 (3%)	5 (3%)	11 (3%)	
Weight, kg	73 ± 12	72 ± 13	73 ± 11	0.637
Height, cm	164 ± 9	163 ± 8	165 ± 9	0.262
BMI, kg/m^2^	26.8 ± 4	26.9 ± 5	26.7 ± 4	0.645
Calorie intake (Kcal/d)	2387 ± 665	2426 ± 724	2365 ± 630	0.320
Carbohydrate %	49 ± 7	49.5 ± 9	49 ± 7	0.883
Protein%	12 ± 2	12 ± 3	13 ± 2	0.287
Fat%	41 ± 10	42 ± 13	40 ± 7	0.040
Total fiber (g/d)	31 ± 14	29 ± 14	32 ± 13	0.007
Total fiber (g/1000 kcal)	13 ± 5	12 ± 5	14 ± 4	0.001
Soluble fiber (g/d)	0.6 ± 0.4	0.6 ± 0.5	0.7 ± 0.4	0.080
Insoluble fiber (g/d)	3.1 ± 2	2.7 ± 1.7	3.3 ± 2.1	< 0.001

Values are means ± SDs for continuous variables and percentages for categorical variables

ANOVA for quantitative variables and χ2 test for qualitative variables

*IPAQ* International Physical Activity Questionnaire, *BMI* Body Mass Index

The subjects’ characteristics and dietary intakes across the tertiles of dietary fiber intake are presented in Table 2. According to Table 2, there were no significant differences in age, alcohol consumption, smoking status, physical activity, weight and BMI between total dietary fiber intake tertiles whereas, significant differences in energy, carbohydrate and fat intakes were found to increase throughout the tertiles.

**Table 2:** Baseline general characteristics and dietary intakes of study participants by tertile of total dietary fiber intake

Total Dietary Fiber Intake				
	Tertile 1 (*n* = 172)	Tertile 2 (*n* = 170)	Tertile 3 (*n* = 171)	*P* value
Cases, n (%)	77 (43)	45 (25)	58 (32)	0.002
Men, n (%)	55 (29%)	57 (30%)	78 (41%)	0.017
Age (y)	54 ± 14	53 ± 13	52 ± 12	0.343
Alcohol drinker	4 (36%)	1 (9%)	6 (55%)	0.173
Smoker, %	26 (37)	18 (26)	26 (37)	0.365
IPAQ level, %				0.079
1	142 (37)	123 (32)	120 (31)	
2	27 (24)	42 (37)	44 (39)	
3	3 (20)	5 (33)	7 (47)	
Weight, kg	71 ± 13	73 ± 11	74 ± 12	0.051
Height, cm	163 ± 8	164 ± 8	165 ± 9	0.132
BMI, kg/m^2^	26 ± 4	27 ± 4	27 ± 4	0.239
Calorie intake (Kcal/d)	2008 ± 551	2333 ± 473	2732 ± 644	< 0.001
Carbohydrate %	47 ± 8	49 ± 7	52 ± ‌ 7	< 0.001
Protein%	12 ± 3	12.5 ± 2	13 ± 2	0.075
Fat%	45 ± 12	42 ± 8	38 ± 7	< 0.001
Total fiber (g/d)	18 ± 4	29 ± 3	47 ± 11	< 0.001
Total fiber (g/1000 kcal)	9 ± 2	13 ± 3	18 ± 4	< 0.001
Soluble fiber (g/d)	0.4 ± 0.3	0.7 ± 0.4	0.7 ± 0.5	< 0.001
Insoluble fiber (g/d)	2 ± 1	3 ± 2	3.6 ± 2	< 0.001

Values are means ± SDs for continuous variables and percentages for categorical variables

ANOVA for quantitative variables and χ2 test for qualitative variables

*IPAQ* International Physical Activity Questionnaire, *BMI* Body Mass Index

Multivariable-adjusted ORs and 95% CIs for gallstone across tertiles of each category of fiber intake were illustrated in Table 3. In the crude model, only insoluble fiber intake showed a significant association with the risk of GSD (OR _T3 vs T1_ = 0.54; 95% CI: 0.3–0.9, P for trend < 0.001). In the age and sex-adjusted model, subjects in the highest tertile of total (OR = 0.45; 95% CI: 0.3–0.7, P for trend = 0.007) and insoluble (OR = 0.53; 95% CI: 0.34–0.84, P for trend < 0.001) dietary fiber intake had lower odds of GSD compared to the first tertile as a reference group. Additionally, in the multivariable-adjusted model, after further adjusting for energy intake, BMI, physical activity, smoking and alcohol consumption, significant reverse associations were observed between odds of GSD and each category of dietary fiber intake including total (OR _T3 vs. T1_ = 0.44, 95% CI: 0.37–0.7, P for trend = 0.015), soluble (OR _T3 vs. T1_ = 0.51, 95% CI: 0.3–0.8, P for trend = 0.048) and insoluble (OR _T3 vs. T1_ = 0.56, 95% CI: 0.3–0.9, P for trend < 0.001).

**Table 3:** Odds and 95% confidence interval for occurrence of the gallstone in each tertile categories of fiber intake

	**Tertiles of fiber intake**			*P* trend
**Total fiber**	T1 (< 24.5)	T2 (24.5–35)	T3 (35 ≤)	
No. of cases	77	45	58	
Model 1	ref	0.63 (0.4–1)	0.49 (0.3–0.75)	0.091
Model 2	ref	0.73 (0.47–1.6)	0.45 (0.3–0.7)	0.007
Model 3	ref	0.65 (0.4–1.3)	0.44 (0.37–0.7)	0.015
**Soluble fiber**	T1 (< 0.37)	T2 (0.37–0.69)	T3 (0.69 ≤)	
No. of cases	98	122	122	
Model 1	ref	0.58 (0.4–0.9)	0.54 (0.2–0.7)	0.081
Model 2	ref	0.55 (0.3–0.9)	0.5 (0.3–0.8)	0.066
Model 3	ref	0.57 (0.3–0.9)	0.51 (0.3–0.8)	0.048
**Insoluble fiber**	T1 (< 2)	T2 (2–3.6)	T3 (3.6 ≤)	
No. of cases	101	116	125	
Model 1	ref	0.69 (0.4–1)	0.54 (0.3–0.9)	< 0.001
Model 2	ref	0.75 (0.48–1.2)	0.53 (0.34–0.84)	< 0.001
Model 3	ref	0.8 (0.4–1.3)	0.56 (0.3–0.9)	< 0.001

Based on multiple logistic regression model

Model 1: crude

Model 2: adjusted for age and sex

Model 3: additionally adjusted for energy intake, BMI, physical activity, smoking, alcohol

**Fig. 1:**
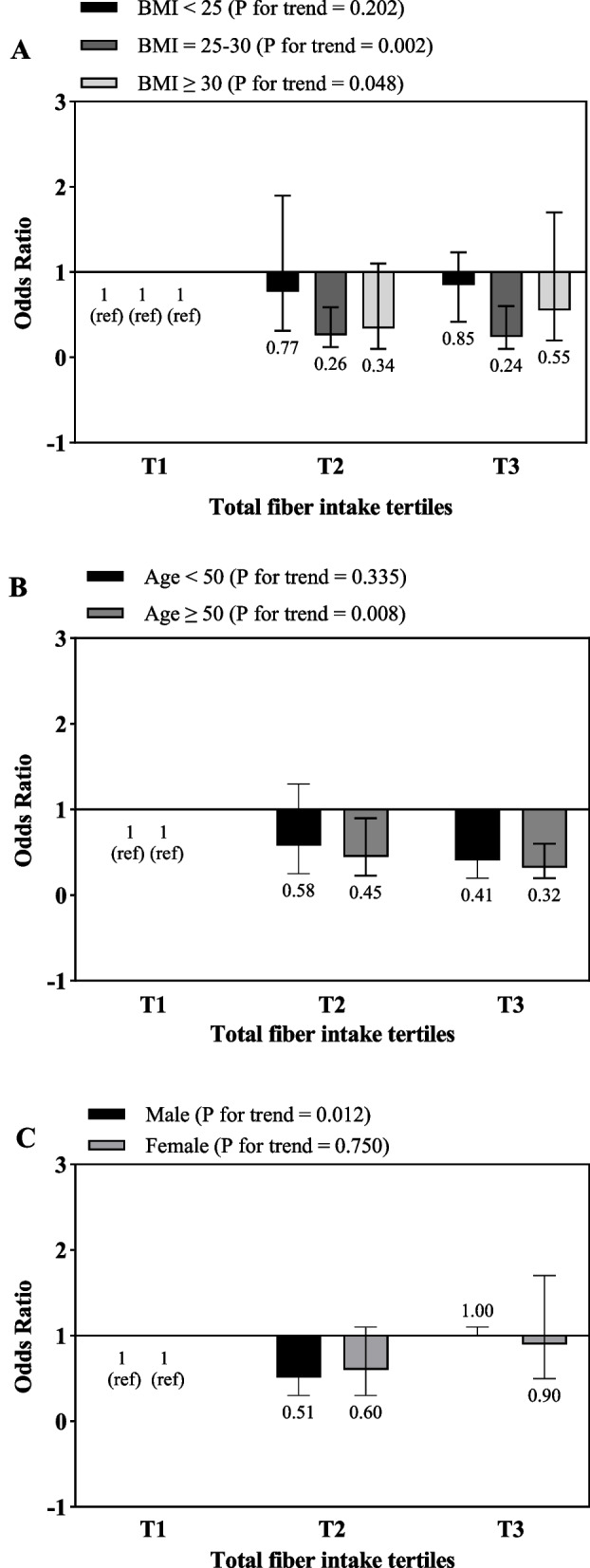
Multivariate odds ratios of total fiber intake tertiles for risk of gallstone according to risk factor status at baseline (multivariate logistic regression models for estimating ORs and 95% CIs, multivariable models were adjusted for sex, age, energy intake, BMI, physical activity, smoking, alcohol, except for the respective stratifying factor). Data are reported as OR (95% CI). **A**, BMI < 25 vs 25–30 and ≥ 30 (*P* = 0.119 for interaction); **B**, age < 50 years vs ≥ 50 years (*P* = 0.711 for interaction); **C**, sex male vs female (*P* = 0.017 for interaction). Ref indicates reference group

**Fig. 2:**
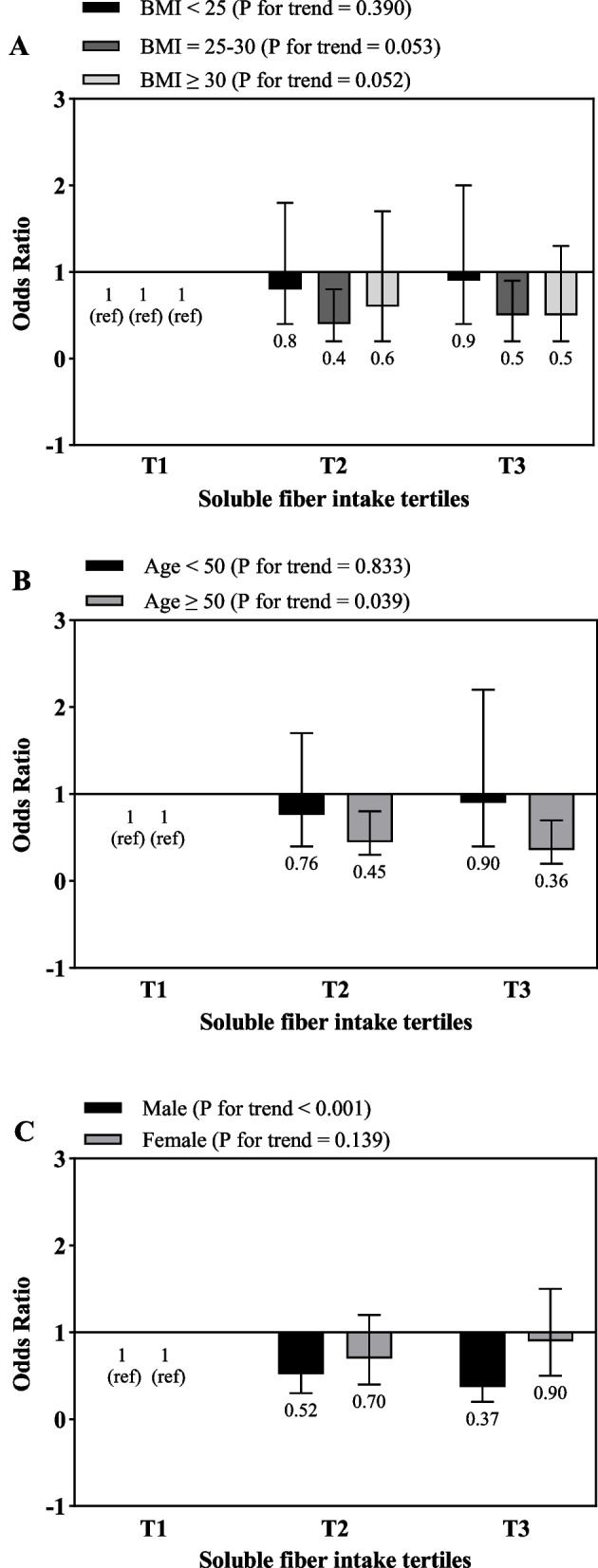
Multivariate odds ratios of soluble fiber intake tertiles for risk of gallstone according to risk factor status at baseline (multivariate logistic regression models for estimating ORs and 95% CIs, multivariable models were adjusted for sex, age, energy intake, BMI, physical activity, smoking, alcohol, except for the respective stratifying factor). Data are reported as OR (95% CI). **A**, BMI < 25 vs 25–30 and ≥ 30 (*P* = 0.119 for interaction); **B**, age < 50 years vs ≥ 50 years (*P* = 0.711 for interaction); **C**, sex male vs female (*P* = 0.017 for interaction)

**Fig. 3:**
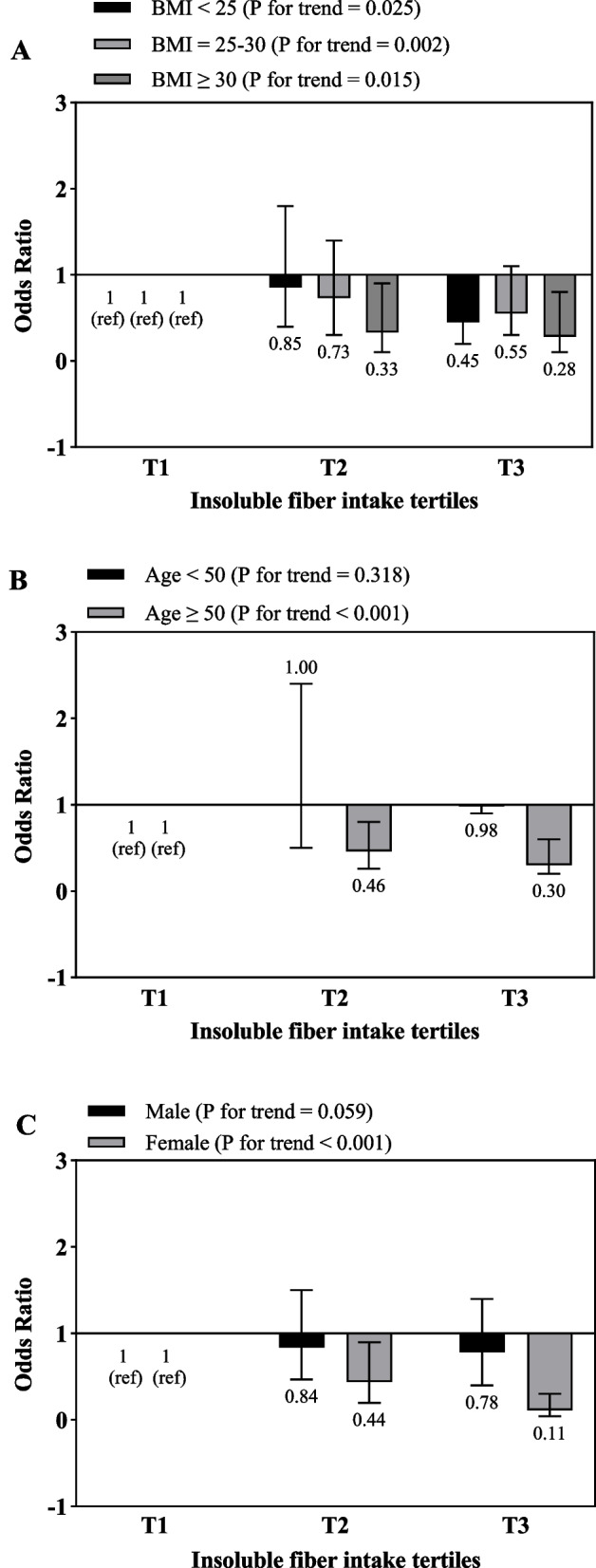
Multivariate odds ratios of insoluble fiber intake tertiles for risk of gallstone according to risk factor status at baseline (multivariate logistic regression models for estimating ORs and 95% CIs, multivariable models were adjusted for sex, age, energy intake, BMI, physical activity, smoking, alcohol, except for the respective stratifying factor). Data are reported as OR (95% CI). **A**, BMI < 25 vs 25–30 and ≥ 30 (*P* = 0.138 for interaction); **B**, age < 50 years vs ≥ 50 years (*P* = 0.581 for interaction); **C**, sex male vs female (*P* = 0.356 for interaction)

Multivariate odds ratios of total, soluble and insoluble fiber intake tertiles for risk of gallstone according to risk factor status at baseline are presented in Figs. 1, 2 and 3, respectively. According to Fig. 1, after adjusting for potential confounders, a significant relationship between total fiber intake and the risk of gallstones was found only in patients who were overweight (OR _T3 vs. T1_ = 0.24, 95% CI: 0.1–0.6, P for trend = 0.002) or obese (OR _T3 vs. T1_ = 0.55, 95% CI: 0.2–1.7, P for trend = 0.002), while, this relationship was not observed in people with normal BMI. Also, this relationship was significant in men and in people over 50 years old.

As shown in Fig. 2, a significant reverse association was observed between the highest and lowest tertiles of soluble dietary fiber intake and odds of GSD in subjects over 50 years old (OR = 0.36; 95% CI: 0.2–0.7, P for trend = 0.039) and male gender (OR = 0.37; 95% CI: 0.2–0.8, P for trend < 0.001).

Figure 3 indicates that subjects in the highest tertile of insoluble dietary fiber intake with age above 50 (OR = 0.3; 95% CI: 0.2–0.6, P for trend < 0.001) and female gender (OR = 0.11; 95% CI: 0.04–0.3, P for trend < 0.001) had lower odds of GSD compared to the reference group. Also, this association was significant for all BMI categories.

## Discussion

To the best of our knowledge, the association of dietary total, soluble and insoluble fiber intake with the risk of gallstone has not been investigated yet. Comparing the highest versus the lowest tertile in the present case–control study showed that dietary total, soluble and insoluble fiber intake were associated with 56%, 49% and 44% lower GSD risk, respectively, after fully adjustment for potential confounders.

These finding are in line with several previous studies. Schwesinger et al. [17] have shown the protective effect of dietary soluble fiber against cholesterol gallstone formation. Two other observational studies have illustrated the inverse relationship between dietary fiber intake and the prevalence of gallstones [18, 19]. Another study investigating the effect of diet as a risk factor for cholesterol gallstone disease implicated that lower and higher intake of dietary fiber and refined sugar, were associated with propensity of gallstone formation, respectively [20]. In addition, consistent with our findings, a large number of epidemiological studies have reported an inverse association between insoluble dietary fiber and GSD [21–24].

In general, by decreasing the intestinal transit time, dietary fibers may reduce the persistence of bacteria located in the colon, which leads to a decrease in the production of secondary bile acids such as deoxycholate, and subsequently, less bile acids are absorbed [10, 25]. Lithogenicity of bile seems to be increased by deoxycholate, whereas chenodexycholate has contrary effect and thus it is used as a therapy to destruct gallstones [26]. Increasing the absorption of deoxycholate can stimulate biliary cholesterol saturation [27, 28]. This claim has been proved in an animal study conducted by Schwesinger et al., [17] who showed that fiber supplementation can prevent the formation of gallstones in prairie dogs on a lithogenic diet.

According to our findings, dietary fiber has the protective effect against GSD especially in older subjects and overweight and obese subjects. These findings are consistent with other published studies considering age and obesity as risk factors for GSD. Based on Masserat et al.’s [3] aging is an important factor leading to gallstone formation among Iranian. Moreover, higher BMI considered as an important risk factor for GSD [29, 30]. Additionally, we showed that women in the highest tertile of insoluble dietary fiber intake had the lowest risk of GSD. According to several studies, female gender is a possible risk factor for GSD [31, 32]. Thus, it seems that the protective effects of dietary fiber intake are more significant in people with related risk factors.

However, it is highly important to note that diets with low fiber content are usually accompanied by higher carbohydrate or/and fat intake, so the effect of fiber on GSD cannot be investigated independently [20]. There are some strengths attributed to current research. Contrary to previous studies, the current study has evaluated all types of biliary stone such as gallstone, common bile duct stone and the history of cholecystectomy during the last six months. Moreover, enrollment of newly diagnosed subjects declined the recall bias. The same interviewer proceeded the study so that the interviewer bias did not happen. Although, we had some limitations as well. First, due to the case–control design of the present study, it was not possible to show a causal relationship between dietary fiber intake and GSD. Second, due to the retrospective nature of the FFQ, the probability of recall bias should be considered. In order to be able to generalize the results, further research needs to be conducted, on a larger scale.

## Conclusion

In conclusion, according to the findings of the current study, dietary fiber intakes have an inverse relationship with the risk of GSD.

## Data Availability

The datasets analyzed in the current study are available from the corresponding author on reasonable request.

## Abbreviations

ANOVA: Analyze of variance
BMI: Body mass index
CI: Confidence interval
FCT: Food composition table
FFQ: Food frequency questionnaire
HRs: Hazard ratios
IPAQ: International physical activity questionnaire
GSD: Gallstone disease
METs h/d: Metabolic equivalents hour per day
OR: Odds ratio
SD: Standard deviation
SPSS: Statistical Package Software for Social Science
USDA: United States Department of Agriculture
